# Accurate prediction of breast cancer survival through coherent voting networks with gene expression profiling

**DOI:** 10.1038/s41598-021-94243-z

**Published:** 2021-07-19

**Authors:** Marco Pellegrini

**Affiliations:** Institute of Informatics and Telematics (IIT), CNR, 56124 Pisa, Italy

**Keywords:** Breast cancer, Machine learning

## Abstract

For a patient affected by breast cancer, after tumor removal, it is necessary to decide which adjuvant therapy is able to prevent tumor relapse and formation of metastases. A prediction of the outcome of adjuvant therapy tailored for the patient is hard, due to the heterogeneous nature of the disease. We devised a methodology for predicting 5-years survival based on the new machine learning paradigm of *coherent voting networks*, with improved accuracy over state-of-the-art prediction methods. The ’coherent voting communities’ metaphor provides a certificate justifying the survival prediction for an individual patient, thus facilitating its acceptability in practice, in the vein of explainable Artificial Intelligence. The method we propose is quite flexible and applicable to other types of cancer.

## Introduction

Breast Cancer is one of the main causes of death in Europe, the USA, and China. The number of new cases each year in Europe is about 92.2 women every 100,000 women. The mortality rate in Europe is 23.1 women every 100,000^1^ (https://encr.eu/sites/default/files/factsheets/ENCR_Factsheet_Breast_2014.pdf).

It is estimated that for 2020 in the US the expected number of new cases of Breast Cancer (BC) in female patients is about 276,000 (30% of all new tumor cases in female patients) and the expected number of deaths caused by Breast Cancer in female patients is about 42,000 (15% of all deaths due to tumors in female patients), thus making BC the first type of cancer for the number of new cases, and the second type of cancer as the cause of death^2^ in female patients. Similar rankings are observed in Europe^3^ and China^4^.

Primary cancer treatment for new cases of BC is surgery (of various types), followed by adjuvant therapies (see e.g.: https://www.gov.uk/government/publications/chemotherapy-radiotherapy-and-surgical-tumour-resections-in-england/chemotherapy-radiotherapy-and-surgical-tumour-resections-in-england). For a patient affected by breast cancer, after tumor removal, it is necessary to decide which adjuvant therapy can prevent the tumor relapse and the formation of metastases. To this effect, a series of measurements of several parameters (clinical, histological, molecular) are collected and evaluated by experts with the help of guidelines.

Conventional clinical–pathological parameters have been used since the definition of the first cancer staging systems in 1946^5^ up to the recent St. Gallen Consensus^6^ to select patients eligible for adjuvant treatment following BC surgery, thus helping in avoidance of unnecessary cytotoxic treatments. The high social and personal cost of chemotherapy and the evidence of over-prescription with the standard methodologies^7^, fueled the search for scientific and technological advances in this area, that could impact clinical practice.

The need for better prognosis and prediction of therapy results has led to substantial research in alternative bio-markers based on BC molecular profiling, and novel prediction models and algorithms, that could overcome intrinsic limitations of previous approaches. In particular high-throughput sequencing technologies have been key enablers for the success of this new approach, as well as the efforts for systematic collection of molecular data.

At this moment prognostic tools based on molecular biomarkers are considered valid clinical decision support tools, complementing traditional histopathology (see e.g. the Mammaprint and Oncotype DX tests)^8^.

Prognostic molecular tests are cost-effective versus the cost of chemotherapy for patients who would not eventually benefit from it. They are considered complementary to histology-based more traditional methods (e.g. TNM staging).

Van’t Veer and her co-authors^9^ describe a panel of 70 mRNA biomarkers for breast cancer predicting survival after 5 years from breast cancer surgery. This panel is the basis for the *Mammaprint* test, which after several clinical trials, has been approved by regulatory agencies in the USA and Europe for clinical use.

Paik et al.^10^ proposed a panel of 16 genes (plus 5 control genes) whose expression level is the basis for computing a score that allows classifying patients into low, medium, and high risk of relapse within 5 years after surgery. This panel is commercialized as *Oncotype DX * and it has been validated in the clinical trial TAILORx^11^. In published data, the intermediate class, which is rather neutral for clinical decisions, covers 30% of the patients in the testing cohort. Other methods for multigene based prognosis of breast cancer are covered in a survey by Győrffy et al.^12^.

In this paper, we describe a novel machine learning (ML) supervised classification method and we apply it to the task of producing prognostic predictions of survival at 5 years for BC patients using gene expression levels measured from the samples of the tumor surgically removed. The prediction method is conditional on the type of post-operative adjunct therapy selected for the patient. Data from a cohort of about 2000 patients available through the Metabric consortium^13^ are used to train, validate and test the prognostic predictor, and they indicate competitive performances compared to state-of-the-art methods. See “Methods” section (Tables 10, 11) for basic statistics of the main features of the populations used for training, validation, and testing.

Survival analysis aims at modeling and estimate complex objects like the *survival function*, or the *hazard function* that give deep insight into the expected survival time (as a continuous function). Here we aim at a more restricted type of result, where survival is dichotomized into low-risk and high-risk classes, with a threshold set at 5 years. The 5 years threshold is a common benchmark in Breast Cancer studies, however, as BC patients often experience a long survival time, also a 10 years benchmark is commonly found in the clinical and epidemiological literature.

*Coherent Voting Network* (CVN) is a supervised learning paradigm designed explicitly to uncover non-linear, combinatorial patterns in complex data, within a statistically robust framework. Breast Cancer patients after surgery may receive several types of post-surgery adjuvant therapeutic regimen (endocrine, radiotherapy or chemotherapy, and combinations thereof) aiming at reducing relapse and the formation of metastases, and thus favoring long term survival. We wish to predict the outcome of adjuvant therapy using just small molecular fingerprints (mRNA) of the patient’s transcriptome. We aim at simultaneous high scores for PPV (positive predictive value) and NPV (negative predictive value) as these are important indices for the final clinical applications of the predictor. A Training-validate-test protocol is applied onto CVN built on patient data from the Metabric Consortium (about 2000 patients).

The performance in tests is at the state-of-the-art for several BC cancer sub-types and it is remarkable for the subclasses: TNBC, Her2+, and Luminal B. The effectiveness of the selected fingerprints is confirmed also on several independent data sets (for a total of 601 patients) from the NCBI Gene Expression Omnibus (GEO).

This article is organized as follows. In “Results”, “Therapy classes” and “Secondary stratifications”, we describe the main results in the application of the CVN-based prognostic predictor on Metabric data. In “Comparison of CVN with other ML classification methods” we compare the CVN-based prognostic predictor against other state-of-the-art ML methods using the *Autoweka* package. In “Performance of CVN on independent cohorts of patients”, we apply the molecular fingerprints derived for Metabric to several independent cohorts of patients. In “Discussion”, we place our results in the context of the currently known results and we comment on strong and weak points of the proposed method, as well as on possible extensions. In “Methods” we give a high-level description of the CVN method and of the data preprocessing, while more details are in the Supplementary Materials.

## Results

### Therapy classes

Patients after surgery may or may not follow one of the following adjuvant therapies: chemotherapy, radiation therapy, and hormone therapy (also called endocrine therapy), which are reported in Metabric annotations. There are thus 8 possible combinations of three therapies. For each therapy profile, we repeat the training-validate-testing procedure to obtain 8 therapy-specific gene sub-panels and prediction performance estimates (primary stratification) (see Supplementary materials 1 for a self-contained recollection of the performance measures used in this context). Table 1 reports 5 therapy classes for which Metabric data are sufficiently numerous to estimate the statistical significance of the predicted performance indices, and the automatic hyper-parameter/feature selection optimization converges.

**Table 1:** Performance of therapy-based stratification.

Therapy	Yes-no-yes	No-no-yes	No-no-no	Yes-no-no	Yes-yes-yes
n.p.	43	31	21	13	35
$>5y$	17	14	7	8	8
$<5y$	26	17	14	5	27
n.a.	37	30	21	13	30
Sen.	0.65	0.81	0.66	0.85	0.8
Spec.	0.92	0.78	0.8	0.66	0.84
OR	24.3	16.8	8.0	12.0	21.0
OR p-val	0.0006	0.002	0.11	0.1	0.01
CI-Lo	2.6	2.55	0.96	0.79	1.8
CI-Hi	221	111	66	180	240
Kappa	0.52	0.58	0.44	0.53	0.51
AUC	0.85	0.87	0.77	0.77	0.63
AUC p-val	0.0001	0.0002	0.02	0.06	0.13
lrt p-val	0.02	0.0006	0.06	0.33	0.03
lh	2	2	4	1	3
fp	7	12	17	8	5

Results on test data with automatic hyperparameter optimization and feature (gene) selection. Therapy class labels are (RAD, CHE, HOR). *n.p.* number of patients, *n.a. * number of answers. 95% confidence interval, *lrt p-val* p value for the log rank test, *lh* lookahead number, *fp* fingerprint size.

The number of genes in each fingerprint for the therapy classes ranges from a minimum of 5 to a maximum of 17, with an average of 9.875. Overall 78 distinct genes are used. The selected fingerprints hardy overlap with previously known fingerprints (see Supplementary materials 1).

### Secondary stratifications

Starting from the 5 sub-panels based on the therapy classes (primary stratification), it is possible to define stratifications based on different features (secondary stratification) of the patient. The secondary stratifications do not change the prediction of any single patient but provide a different evaluation of the quality of the prediction. We take into consideration ER status as measured by IHC (Table 2), Intrinsic Type (Table 3), ER/HER2 classification (Table 4), Tumor stage (Table 5), Tumor grade (Table 6), and Lymph node state (Table 7).

**Table 2:** Secondary stratification by ER status.

Type	n.p.	$>5y$	$<5y$	n.a.	Sen.	Spe.	or	p-val	CI-Lo	CI-Hi	Kappa	lrt pval	PPV	NPV
Pos	116	78	38	107	0.67	0.83	9.83	6.67e-07	3.88	24.93	0.50	0.001	0.67	0.83
Neg	24	10	14	21	0.86	0.71	15.00	0.02	1.63	138.16	0.57	0.01	0.86	0.71

**Table 3:** Secondary stratification by intrinsic status.

Type	n.p.	$>5y$	$<5y$	n.a.	Sen.	Spe.	or	p-val	CI-Lo	CI-Hi	Kappa	lrt pval	PPV	NPV
LumA	45	37	8	41	0.25	0.88	2.42	0.58	0.36	16.34	0.14	0.11	0.33	0.83
LumB	41	26	15	37	0.92	0.83	60.00	1.09e-05	5.98	601.61	0.72	0.05	0.75	0.95
Claudin-low	14	7	7	13	0.71	0.83	12.50	0.10	0.84	186.31	0.54	0.24	0.83	0.71
Her2	22	12	10	20	0.90	0.60	13.50	0.06	1.20	152.22	0.50	0.79	0.69	0.86
Basal	14	3	11	14	0.82	0.67	9.00	0.18	0.52	155.25	0.43	0.06	0.90	0.50

**Table 4:** Secondary stratification by 3 genes status.

Type	n.p.	$>5y$	$<5y$	n.a.	Sen.	Spe.	or	p-val	CI-Lo	CI-Hi	kappa	lrt pval	PPV	NPV
her2+	18	8	10	15	1.00	0.80	40.00	0.01	1.98	807.14	0.84	0.01	0.91	1.00
er+/her2−	98	68	30	90	0.57	0.84	6.93	1.37e-04	2.53	19.02	0.42	0.07	0.62	0.81
er−/her2−	16	6	10	15	0.90	1.00	45.00	7.62e-03	2.29	885.65	0.86	0.004	1.00	0.83

**Table 5:** Secondary stratification by tumor stage.

Type	n.p.	$>5y$	$<5y$	n.a.	Sen.	Spe.	or	p-val	CI-Lo	CI-Hi	Kappa	lrt pval	PPV	NPV
1	27	20	7	26	0.71	0.84	13.33	0.01	1.71	103.76	0.53	0.03	0.62	0.89
2	68	43	25	61	0.80	0.78	14.00	1.66e-05	3.99	49.16	0.57	0.009	0.71	0.85
3	13	5	8	13	0.62	1.00	8.33	0.14	0.63	110.03	0.56	0.02	1.00	0.62

**Table 6:** Secondary stratification by tumor grade.

Type	n.p.	$>5y$	$<5y$	n.a.	Sen.	Spe.	or	p-val	CI-Lo	CI-Hi	Kappa	lrt pval	PPV	NPV
2	54	39	15	45	0.77	0.84	18.00	1.75e-04	3.62	89.58	0.59	0.0006	0.67	0.90
3	75	40	35	72	0.74	0.76	8.99	4.38e-05	3.09	26.13	0.50	0.02	0.74	0.76

**Table 7:** Secondary stratification by lymph node status.

Type	n.p.	$>5y$	$<5y$	n.a.	Sen.	Spe.	or	p-val	CI-Lo	CI-Hi	Kappa	lrt pval	PPV	NPV
POS	82	48	34	75	0.70	0.83	11.50	4.19e-06	3.83	34.54	0.54	0.0003	0.77	0.78
NEG	61	41	20	56	0.79	0.81	16.07	2.41e-05	4.06	63.63	0.58	0.005	0.68	0.88

**Figure 1:**
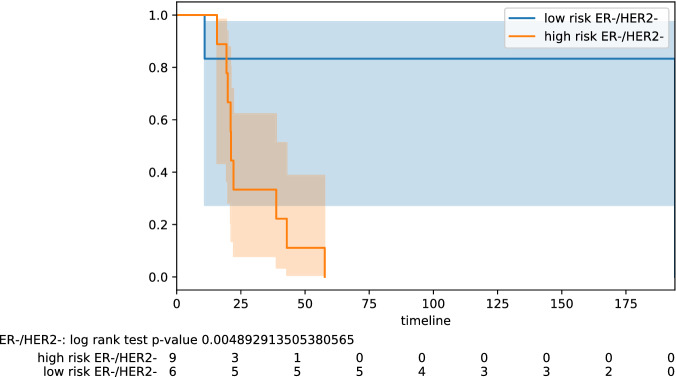
Stratification by hormonal type: ER−/Her2−.

**Figure 2:**
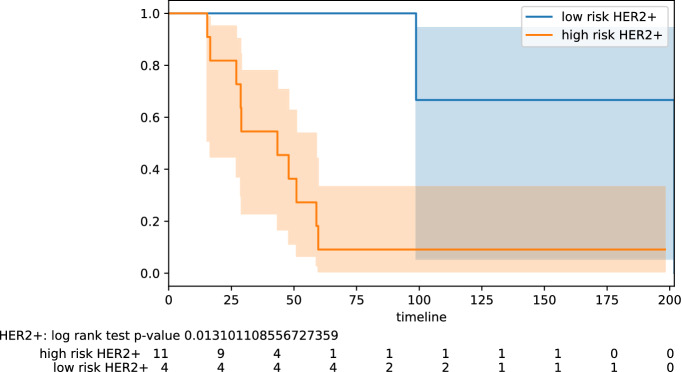
Stratification by hormonal type: Her2+.

**Figure 3:**
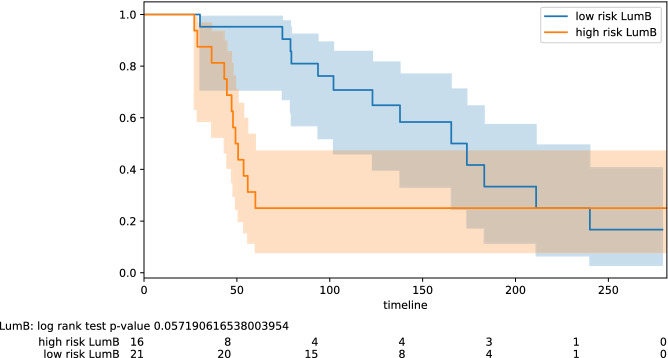
Stratification by intrinsic type: Luminal B.

**Figure 4:**
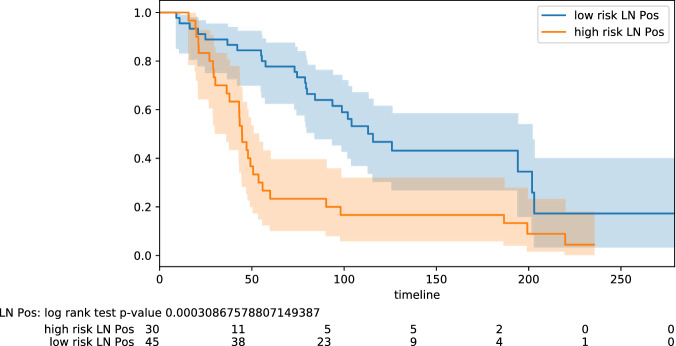
Stratification lymph node status: positive.

**Figure 5:**
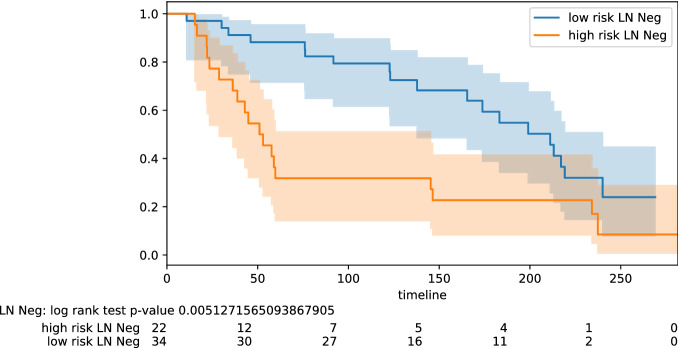
Stratification lymph node status: negative.

Here we highlight some of the tabled results. For the testing pool of 82 lymph node positive patients, we obtain PPV 0.77 and NPV 0.78 (odds ratio 11.50); for the pool of 61 lymph node negative patients, we obtain PPV 0.68 and NPV 0.88 (odds ratio 16.07). Improved results are obtained on some specific subtypes of BC. For the testing pool of 16 TNBC patients, we obtain PPV 1.0 and NPV 0.83 (odds ratio 45.00). For the testing pool of 18 HER2+ patients, we obtain PPV 0.91 and NPV 1.0 (odds ratio 40.00). For the testing pool of 41 Luminal B patients, we obtain PPV 0.75 and NPV 0.95 (odds ratio 60.00). The PPV and NPV results should be seen in the context of the prevalence (the proportion of the population with high vs low risk) in the test sets used. Kaplan–Meier plots for notable subclasses are shown in Figs. 1, 2, 3, 4 and 5. Kaplan–Meier plots for all the secondary stratifications are shown in the Supplementary Materials 1.

### Comparison of CVN with other ML classification methods

To compare our algorithmic solution with the state-of-the-art in machine learning, we performed experiments with the Autoweka package^14,15^ within the Weka workbench environment^16,17^. Autoweka performs automatically feature selection and hyper-parameter optimization of 27 base classification methods, 10 meta-methods, and two ensemble methods, moreover it uses several feature selection search methods along with 8 feature evaluation functions. The hyper-parameters are optimized in Autoweka using a Bayesian optimization strategy to explore the space of parameters. The Autoweka package includes also the Ridge regression method (a form of simple penalized logistic regression ) often used in survival analysis. The ridge parameter is optimized by Autoweka in the range of values from 1e$-7$ to 10.

As we noticed that the initial feature selection phase is onerous when applied to the input of roughly 24,000 genes, we also applied explicitly several Weka feature selection pre-filters so to reduce the number of features in the input to Autoweka. Autoweka uses ten-fold cross-validation over the training set to select the best configuration of hyper-parameters. We fixed the kappa statistics as the objective function to be maximized in the learning phase (see Supplementary Materials 1). The reported kappa statistics are computed on the trained predictor for the test data set.

Table 8 reports the kappa statistics for the best Autoweka trained classifiers (in round brackets) along with the result we obtain with the coherent voting networks over the test set. For CVN next to the kappa statistics we report the lookahead number or, in two cases, the manual selection of the best configuration. Ignoring for the moment the two manually selected configurations, we notice that we can get the highest kappa values in three of the six remaining cases. We also notice for CVN a robust uniform behavior with consistent high positive values of kappa. In all columns (except corr-ranker) there are negative entries indicating that the best ML method for that input has performed worse than a random classifier. For the *corr-ranker* feature selection the ML methods have all positive values, but generally lower than those of CVN. Moreover, the best Autoweka results are attained by 15 different methods thus making it hard to pinpoint a single winner algorithm in the Autoweka suite.

Overall the setup experimental conditions for the Autoweka and CVN differ in some aspects, therefore the findings must be considered with care. Keeping these differences in mind, we can conclude that CVN has a level of performance at least comparable with existing ML methods. Moreover, CVN is a single easy-to-explain method that allows for a more uniform approach to the BC prognosis problem over a wide spectrum of clinical conditions.

**Table 8:** Kappa statistics for training data sets for various Autoweka/Weka feature selection settings.

Therapy	No filter	cfs-best	cfs-greedy	Corr-ranker	Gain-ranker	j48-ranker	j48-greedy	CVN (lh)
Yesnoyes	0.30 (rf)	0.52 (mp)	0.52 (mp)	0.23 (lo)	0.38 (smo)	0.33 (lwl)	**0.58** (mp)	0.52 (2)
Nonoyes	0.35 (sl)	0.34 (nb)	0.15 (rf)	0.22 (smo)	0.16 (lwl)	0.35 (bn)	0.15 (rf)	**0.58** (2)
Nonono	0.09 (rf)	**0.64** (bn)	0.5 (smo)	0.35 (ibk)	0.35 (nb)	0.35 (nb)	0.50 (rf)	0.44 (4)
Yesnono	**0.7 ** (rf)	0.53 (sgd)	0.69 (rf)	0.39 (lo)	0.56 (rf)	0.56 (rf)	**0.7 ** (rf)	0.53 (1)
Yesyesno	− 0.09 (dt)	0.36 (rc)	0.05 (nb)	0.22 (lwl)	0.10 (lwl)	0.0 (ab)	0.05 (nb)	**0.48** (m)
Yesyesyes	− 0.07 (nbm)	− 0.07 (ibk)	− 0.01 (ibk)	0.19 (smo)	0.14 (lwl)	0.26 (rss)	− 0.07 (ibk)	**0.51** (3)
Noyesno	− 0.26 (rf)	− 0.03 (mp)	− 0.03 (mp)	0.11 (mp)	− 0.26 (smo)	− 0.22 (mp)	− 0.03 (mp)	**0.41** (2)
Noyesyes (*)	0.0 (rpt)	− 0.53 (rf)	− 0.53 (rf)	0.13 (rf)	0.17 (rf)	0.23 (rf)	− 0.54 (rf)	**0.60** (m)

Therapy class (RAD, CHE, HOR). *lh* lookahead number or manually determined (m). Legend for autoweka methods: *rf* random forest, *mp* multilevel perceptron, *nb* Naive Bayes, *bn* Bayes Net, *sgd* stochastic gradient descent, *rc* random committee, *ibk* k-nearest neighbour classifier, *sl* simple logistic, *nbm* Naive Bayes Multinomial, *rpt* Fast Decision Tree REPTree (C4.5), *smo* fast training support vector machine, *lo* Logistic, *lwl* Locally Weighted Learning, *ab* AdaBoostM1, *rss* random subspace, *dt* decision table. (*) result for the validation dataset.

### Performance of CVN on independent cohorts of patients

After a screening of the breast cancer data sets in the NCBI GEO (Gene Expression Omnibus) repository we have identified a few BC data sets with characteristics compatible with the Metabric data set regarding the recorded therapy, endpoint survival (preferentially overall survival). The prediction performance is tested in a leave-one-out evaluation framework in which the multi-gene fingerprint is the optimal fingerprint defined on Metabric data. Greedy hyper-parameter optimization is applied and the best result in terms of OR subject to slackness below 15% is the selected configuration reported in Table 9. Due to different microarray technologies, we have mapped the genes onto the probes for the target technology (using all mapping probes, if multiple probes map onto the same HUGO gene ID).

The GSE45255 data set holds information on three different therapeutic classes. The numbers of patients in each class are however rather small. While for the GEO45255 chemotherapy (ch) subset there is perfect performance, the p-value for OR is too high for claiming statistical significance on this measure, but, in contrast, the AUC value is statistically significant. For the GEO45255 endocrine (ho) and the GEO45255 endocrine plus chemotherapy (chho) subsets, we attain high values in kappa and OR, with significant OR and AUC p values.

Data set GSE37181 holds a large number of patients (119), and it is perfectly balanced among the two classes (60 vs 59), but the endpoint is disease-free survival (dfs), rather than overall survival (os). We notice a loss in terms of OR although the kappa statistics and AUC are still in an acceptable range, with good statistical significance.

Data set GSE7390 holds a larger number of patients (181) but is unbalanced among the two classes (157 vs 24). This has the effect of inducing relatively low kappa statistics, however, the odds ratio (20.86), sensitivity (0.7), specificity (0.89), and the p values indicate a good performance on these indices.

Data set GSE2034 is the largest independent cohort (264) in this table and is roughly balanced (95 vs 169) within a factor 2. Although the kappa statistics is low, the odds ratio OR is high (20.12) even if the endpoint is relapse-free survival (rfs) rather than overall survival (os).

Overall these experiments show that the selected multi-gene fingerprints may be effective across different microarray platform and different patient cohorts, while some loss of performance can be expected when a different endpoint is used. This suggests that when we change the endpoint of the prediction (e.g. disease-free survival) we should recalibrate the fingerprints in the chosen setting.

**Table 9:** Independent cohorts.

GEO	45255 (ch)	45255 (ho)	45255 (chho)	37181	7390	2034
End point	os	os	os	dfs	os	rfs
Therapy	No-yes-no	No-no-yes	No-yes-yes	No-no-no	No-no-no	Yes-no-no
n.p.	8	16	13	119	181	264
$>5y$	3	6	4	59	24	95
$<5y$	5	10	9	60	157	169
n.a.	8	16	13	106	179	258
Kappa	1.0	0.58	1.0	0.35	0.36	0.12
Sen.	1.0	0.8	1.0	0.66	0.7	0.90
Spe.	1.0	0.81	1.0	0.70	0.89	0.67
OR	15.0	18.0	36.0	4.59	20.86	20.12
CI-Lo	0.66	1.24	1.77	2.01	4.93	2.53
CI-Hi	339	260	731	10	88.2	159
OR p-val	0.19	0.03	0.01	3.7E−4	3.0E−5	1.8E−4
AUC	1.0	0.89	1.0	0.72	0.70	0.63
AUC p-val	0.01	0.005	0.003	2.6E−5	7.5E−4	1.0E−4

Results of leave-one-out evaluation with optimal multigene fingerprints derived from Metabric data sets. Therapy class: (RAD, CHE, HOR). Endpoint (e.p.) is *os* overall survival, *dfs* disease-free survival, *rfs* relapse-free survival. Confidence interval for odds ratio at 95% confidence. *n.a*. number of answers.

## Discussion

We have developed a new ML supervised classification method called *Coherent Voting Networks* (CVN) which is suitable for handling highly non-linear phenomena such as those prevalent in biological systems. We have applied CVN to the problem of predicting the prognosis of BC patients depending on the chosen post-surgery adjuvant therapy selected. After surgery, a breast cancer patient must follow a therapeutic regime aimed at preventing relapse and formation of metastases. The CVN-based prognostic tool can predict, with good accuracy for a large percentage of the patients, whether the patient will survive more or less than 5 years following current the state of the art adjuvant therapeutic protocols (based on chemotherapy, radiation therapy, and hormone therapy). Such prognostic tool helps the clinician and the patient by validating the chosen therapeutic path (in case of predicted good prognosis), or by suggesting, in combination with other elements, the need for further investigations, or the application of newer, possibly experimental, alternative protocols (in case of predicted poor prognosis). The advantage for the patient is the possibility to personalize the therapeutic choices by using her molecular prognostic profile, with a higher chance of an effective cure and survival. The advantage for the clinician is a tool to validate baseline therapeutic choices (or suggest the need for alternatives). The advantage for the health system at large is better discrimination among those patients requiring expensive and invasive cures (e.g. chemotherapy), and those that would benefit from less expensive and invasive ones (e.g. hormonal therapy). The CVN-based prognostic tool uses a small molecular profile of a few dozen genes that can be measured for each patient’s tumor biopsy with standard technologies like RNA-seq or RT-PCR.

The fingerprint gene panel has been identified using public data of the project Metabric (Molecular Taxonomy of Breast Cancer International Consortium) and tested using other publicly available data of independent cohorts. Thus the results in this paper rely rather heavily on the quality of the Metabric protocols for collecting molecular and clinical data. An interesting line of research to be developed is to assess the robustness of the CVN-based prediction when different technologies and different data processing protocols are used. Preliminary tests on independent cohorts (see Table 9) suggest that the devised gene fingerprint is rather robust with respect to changes in the gene expression measurement technology and are even capable of operating with endpoints different from the default one chosen in this study (overall survival). However the hyper-parameter optimization phase during predictor’s training is likely to be rather more data and technology-dependent, and thus probably the adoption of different technology/protocol in data collection may entail a re-training of the predictor. A second limitation of the method in its training phase is that it relies on knowledge of the adjuvant therapy chosen for the patients. There is an implicit assumption that over the time frame of the data collection no drastic changes in the clinical practice and criteria would take place. As this cannot be guaranteed over a long period (and indeed changing current clinical protocols is the final aim of this tool) there is the practical need of continuous monitoring to ensure consistency between the patient population used in training and the population for which the tool is applied.

The CVN methodology is a general ML supervised classification tool, and, for prognostic purposes, it can be in principle applied to many variants of this problem.

The CVN-based prognostic tool is currently optimized to maximize and balance the kappa statistics (alternatively the odds ratio) across training, validation, and test data while limiting the number of patients for which no answer is given. This strategy produces also often a balancing of PPV and NPV. It is possible to obtain alternative gene panels for a specific situation (or different predictors on the same panels) that may optimize directly PPV and NPV, say by maximizing PPV subject to a lower bound on NPV (or vice versa).

Also, when a higher rate of no-answers is allowed we can increase the PPV and NPV for the given answers. Preliminary data for certain therapy classes give an NPV and PPV close to 95% for 50% of the patients. Thus with the same data, it is possible to devise a cascade of predictors having higher guarantees for the easier cases, as to cover a given population by several stratified predictors (from the easiest to the most complex cases to predict).

It is possible in principle to apply this CVN methodology to derive a prognostic panel at 10 years (this information has also clinical relevance in long-term follow-ups).

In general, it should be possible to derive similar gene panels for other tumors, provided that Metabric-like high quality data is available on a sufficiently large cohort of patients.

Finally, since we have used only gene expression data (and knowledge on the patients 5-year survival) to build the predictors, one may think that feeding other clinical or molecular indices as additional input to the CVN may improve the predictive powers. Preliminary experiments in this direction however show that a straightforward integration of known single clinical measurements does not improve predictions significantly. It remains thus open the question whether more sophisticated heterogeneous data integration strategies taking several indices at once may be beneficial within the CVN approach to prognosis predictions. A promising line of future research involves integrating mRNA and miRNA to produce mixed prognostic signatures^18,19^. Data on miRNA expression in Metabric patient’s samples have been produced recently within the *Metabric miRNA landscape project* (https://ega-archive.org/studies/EGAS00000000122). Preliminary results from this project indicate that “breast cancer miRNAs appear to act as modulators of mRNA-mRNA interactions rather than molecular switches”. Thus while it is likely that mixed miRNA-mRNA fingerprints may sharpen some of our results, within the CVN framework, we expect that mRNA will continue to be key elements of the predictors, even in this extended setting. Certainly, a better appreciation of miRNA-mRNA interactions in BC may shed more light on the causative elements of BC progression. A second promising direction of research integrates biomedical imaging and molecular profiling for prognostic purposes^20,21^.

Triple-negative breast cancer (TNBC) is an aggressive type of breast cancer affecting about 15% of the cases, and it is known to be quite non-homogeneous from a clinical and molecular point of view^22–24^. Research on devising prognostic molecular fingerprints for TNBC has thus been directed mainly at subclasses of of TNBC^25–31^. In our results, we are able to attain good performance in terms of PPV, NPV, and OR on the full pool of Metabric TNBC patients. The good overall performance may be explained with the intuition that the initial therapy-based stratification of the patients is able to capture implicitly the TNBC molecular and clinical heterogeneity.

HER2 positive BC covers about 25% of the BC cases. It is considered an aggressive tumoral form, and while it responds well to recent therapeutics, it is known to develop drug resistance in time for about 50% of the cases distant metastases occur^32–35^. Molecular signatures for HER2-positive BC prognosis have been found for certain subtypes of the disease or for predicting the response to specific drugs^36–39^. Also for this important type of BC, we could attain high PPV, NPV, and OR results.

Lumina-B BC is one of the intrinsic types of BC discovered by Perou et al.^40^, based on clustering of BC gene expression profiles. Prognostic properties of this subtype have been investigated in particular compared to the other intrinsic types^41,42^. In general, however, less is known about discriminating prognosis within the type^43,44^. Here we show that the CVN-based classifier is effective in discriminating good and poor prognosis patients with high PPV, NVP, and OR.

van de Vijver et al.^45^ report the performance of a 70-genes prognostic gene fingerprint: for lymph node negative patients OR is 15.0 (3.3–56, p val < 0.001) with PPV 0.63 and NPV 0.89. For lymph node positive patients OR is 13.7 (3.1–61 , p val < 0.001), with PPV = 0.4 and NPV 0.95. Overall our results for lymph node positive and negative are similar in terms of OR but, in our case, we have a better balancing between the PPV and NPV measures.

Paik et al.^10^ developed a 21-gene signature (16 predictive and 5 control genes) to predict recurrence in lymph node negative breast cancer treated with Tamoxifen, which was later incorporated in the Oncotype DX prognostic kit. Taking into account only the low and high-risk classification of the patients we obtain an OR 5.67 (3.39–9.46, p value 9.6e−12) with NPV 0.90 and PPV 0.38. Again, our results show a better balancing of PPV and NPV values.

Our work has focussed on selecting relatively small fingerprints that can be used to build predictive CVN, by maximizing the kappa statistic (or the odds ratio) in testing sets of patient data, subject to an upper bound on the slackness of the method (percentage of no responses). In this research, we did not aim at uncovering *causative* fingerprints (i.e. a pattern of gene expression level measures that *explain* the future survival in combination with a therapeutic regime^46^. Although we cannot rule out that the uncovered genes may indeed be involved in the causation of the disease, two orders of considerations advise caution. One consideration is that several just slightly sub-optimal fingerprints may also be found (a phenomenon compatible also with the findings by Venet et al.^47^). Thus causative genes may be present outside a predictive fingerprint of minimal size, with an explanatory role as important as that of those present in the fingerprint. The second consideration is that we have used one mRNA data set from protein-coding genes as our feature space. It is known that BC involves several layers of biological regulation (e.g. genetic aberrations, actions of non-coding RNA, epigenetic signals, multi-cell signaling, metabolic and environmental conditions), thus a causative explanation might involve a more complex interplay of several layers. Finally, we did not touch yet on the topic of whether such fingerprints contain directly actionable targets for therapeutic agents (either for administered drugs or for new drugs tailored to the personal molecular profile of the patients). These related problems are of interest and may entail the collection and fusion of additional relevant ‘omic’ data, as well as the refinement of the algorithms introduced in this study.

## Methods

Here we give an overview of the Coherent Voting Network (CVN) methodology at a high level. For details, we refer to the Supplementary materials 1 (“Methods” in detail). The description is in two parts. The first part introduces the CVN and its use for prognosis prediction. The second part describes the feature-selection and hyper-parameter optimization procedure that is performed in a train-validate-test protocol aiming at optimizing the gene fingerprint, the CVN configuration, and estimating the performance of the method on a testing set of patients.

### Construction of a CVN

As working with a complete gene set is a computational burden and may introduce too much noise from the experimental measurements, we apply a mild initial statistical filter to preserve in the computation only genes able to discriminate the two categories of patients (high-risk or low-risk) that correspond to bad and good prognosis, using thresholds for fold change, t test, ks-test (Kolmogorov–Smirnov), and mwu-test (Mann–Whitney U). Thus the gene set we use in the further CVN construction is composed of genes passing a combination of these statistical discrimination tests.

We build a bipartite graph *G* in which we have patient nodes *P* and Gene-Interval nodes *GI* where each node of the *GI* class is labeled with a gene and an interval of values for the expression of that gene. This graph is built in a straightforward manner from the input data matrix of gene expression for a pool of patients, by using quantization methods^48^. We build a partial dense cover of this bipartite graph (see definition in Pellegrini et al.^49^) which is a collection ${\mathcal {C}}$ of dense subgraphs of *G*, where each subgraph is also called a *community*. Each community will have both patient and gene nodes, and the communities may overlap. Let us for the moment concentrate only on the patient nodes. Each patient may belong to many communities. Each patient has a category (high-risk or low-risk) that corresponds to a bad or good prognosis. Each community expresses a vote (high-risk, low-risk, or null) by a voting scheme (say, for the moment, simple majority, but more schemes are described in the Supplementary Materials 1). Each patient receives a prediction that is the majority category expressed by the communities it belongs to. Finally, the voting is coherent for a given patient *p* if the vote received by *p* is equal to her category. The degree of coherence of the voting network is the fraction of patients for which it is coherent. Ideally, the higher the degree of coherence of a CVN the better such CVN is as a basis for a predictor. The key point is that in such a construction the partial dense cover does not depend on the category of the patients, thus we may have in input non-classified patients, for which the vote of the network represents their category prediction. The intuition is that a network that is coherent for the classified patients, even if built without knowing their category, is a good predictor also for the unclassified ones.

We can see a CVN as a generalization of the notion of *guilt by association* (GbA) in biological networks. In a typical application, some nodes in a biological network will have labels and some will be unlabeled. We make a prediction for an unlabeled node by using the labeled nodes within a neighborhood of the unlabeled node in the biological graph. Note that in GbA each node receives a vote from a *single subset* of the nodes.

So far each community in a CVN may have a large number of genes, and one of our aims is to find a minimal set of genes that leave the communities (of patients) unchanged since the reduction of the number of genes would not change much their density. To achieve this goal we consider now only the genes belonging to any community. We look for a minimal set *M* of genes so that each community (of genes) includes at least *k* genes in *M*. The set *M* can be well approximated by using a greedy set multi-cover algorithm (see e.g.^50^).

After computing the minimal set *M* of genes we can rebuild the CVN using only the patient set *P* and the genes in *M* obtaining a CVN’, measure the coherence of CVN’, and use CVN’ for prediction of the category of unclassified patients.

### Train-validate-test protocol

Each phase of the construction described above depends on the choice of values for hyper-parameters, and we will have a CVN for each such choice (which we call a parameter-vector *v* of the parameter-space *V*). While sophisticated strategies for searching this discrete parameter-space exist (in ML they are termed *hyper-parameter optimization strategies*) in our application the construction of a single CVN is in practice very efficient thus we will use *greedy search* and compute a CVN for each $v \in V$, as |*V*| is in the range of only a few hundreds.

A further aim, besides finding an optimal *v*, and a small gene set *M* is to have high performance for the testing phase in a train-validation-test set-up.

We begin by splitting the initial set of patients into three sets: the training set *T*0, the validation set *T*1, and the test set *T*2. In a standard ML setting information leaking is avoided by finding the optimal $(v^*,M^*)$ pair only on (*T*0, *T*1) and then applying such optimal predictor to (*T*0, *T*2). The performance is measured on this unique predictor for (*T*0, *T*2). We relax such an all/nothing schema by allowing the use of *T*2 in the choice of $(v^*,M^*)$ in a very limited and controlled way, by use of the concept of *lookahead*. Instead of producing a single predictor on (*T*0, *T*2) we produce a ranking of all predictors on (*T*0, *T*1) that we can build by choosing a $v \in V$. We then lookup vectors *v* in this ranked list, and we stop when the corresponding predictor for (*T*0, *T*2) satisfies a stopping criterion. The number of vectors *v* we visit in this lookahead process is the *lookahead number* (lh). For lh=1 we have the standard ML set up. In Table 1 we report the lh values observed for the therapy classes: 1 once, 2 twice, 3 once, and 4 once.

### Computation of p values

In this study, we use three different p values associated with the statistics: odds ratio, ROC AUC, and log-rank test. The log-rank test statistic and the associated p value are computed with the API of the *lifelines* package (https://lifelines.readthedocs.io). The log-rank test statistic is a chi-squared test under the null hypothesis of the two series having the same hazard ratio. The ROC AUC statistic and the associated p value are computed via equivalence to the Wilcoxon Mann Whitney test using the API in the *scipy.stats* package (https://scipy.org/). This p value is one-sided and assumes an asymptotic normal distribution. The Odds Ratio statistic and the associated p-value are computed with the exact Fisher test API in the *scipy.stats* package, with the ‘two-sided’ option. In this test, the distribution of odds ratio values follows the hypergeometric distribution.

### Metabric patients sample selection

The Metabric collection used in this paper is described in Curtie et al.^13^ and is made of 1992 clinically annotated primary fresh-frozen breast cancer specimens from tumor banks in the UK and Canada. The Metabric collection is the union of two main cohorts: the first cohort of 997 female patients and the second cohort of 995 female patients. The differences among the two main cohorts do not impact our research, as the relevant transcriptomic methodology adopted is the same in both cases, thus we do not distinguish the cohort of origin in subsequent steps. Note also that we do not use data on the normal tissue specimens.

Nearly all estrogen receptor (ER)-positive and/or lymph node (LN)-negative patients did not receive chemotherapy, whereas ER-negative and LN-positive patients did. Moreover, none of the HER2+ patients received trastuzumab. Thus the treatments were homogeneous within clinically relevant groupings, which is an important feature of this collection supporting our therapy-based stratification. Although many assays are available for this collection of specimens, here we exploit the clinical data only in conjunction with the normalized gene expression matrix.

All patient specimens were obtained with appropriate consent from the relevant institutional review boards^13^. RNA was isolated from samples and hybridized to Illumina HT-12 v3 platforms for transcriptional profiling. Illumina HT-12 v3 technology targets more than 25,000 annotated genes with more than 48,000 probes. Probes were designed using the RefSeq (Build 36.2, Rel 22) and the UniGene (Build 199) databases. Illumina HT-12 v3 raw data is then preprocessed in steps: spatial artifact correction, summarization, normalization of Log2 intensities with *beadarray* (https://bioconductor.org/) and *bash*^51^ (see Suppl. Mat. in Curtis et al.^13^).

Each patient is annotated with her risk class, taking censoring into consideration, setting survival below 60 months (5 years and below) as high-risk, and survival above 72 months (6 years and above) as low-risk. We take the full collection of 1992 Metabric patients in a vector and apply a random permutation (function random.shuffle in python). Next, we assign the first half of the positions of the vector to the training (1000 patients), the subsequent quarter of the positions to validation (500 patients), and the last quarter to testing (492 patients). Note that due to the properties of random permutations every subset of patients of the corresponding size has the same chance of showing up as a training/validation/testing set. We perform also on the three sets an equalization step. Within the majority risk-class, we take a random sample (via random permutation) of the same size as the minority risk-class. The patients in the majority risk class not selected are discarded. Afterward, each of the three sets (training/validation/testing) in the two variants (unequalized/equalized) is stratified according to the eight possible therapy classes, as reported in the clinical annotations. When for a therapy class the two risk-classes groups are sufficiently balanced in the unequalized case for the three sets (with a ratio below 2.5:1 of the largest class to the smallest) we use the unequalized sets. Otherwise (unbalanced case) we use the equalized versions of the sets.

### Handling missing data

When data is used in matrix form missing data need to be taken into account before numerical computation may start, as a fully dense matrix is usually assumed by most numerical methods. In our setting data is presented in a matrix, and re-mapped to a graph G after the application of the initial statistical filters and discretization. Initial statistical filters and discretization are done in a gene-by-gene fashion and they are well-defined operations even when some matrix entries are missing. Here we just apply loose filtering, excluding by default genes having more than 50% of missing entries. For the phase of graph generation missing entries in the matrix are simply mapped to missing edges in the graph. Note that, from a formal point of view, graph algorithms do not suffer from this. More specifically in our context, missing edges may result in lower density for the communities associated with the incident nodes, and this situation is handled in full generality by the algorithm for building the dense communities. Our approach thus avoids altogether any potential bias or noise introduced by the standard missing data handling methodologies that rely on interpolation.

### Handling censored data

Our approach to dichotomization and censoring can be classified as an “uncensoring” technique^52^, a transformation of the input data so that standard classification algorithms can be applied effectively to censored data. Our approach has similarities with the method described in Zupan et al.^53^, where the instances in the given data are split into three categories: (1) instances that experience the event of interest (death by BC related causes) during the observation period will be labeled as eventful and assigned to the risk class according to the event time; (2) instances whose censored time is later than the predefined time point (5 years) are assigned to the low-risk class; (3) instances whose censored time is earlier than the predefined time point are removed from further consideration. In the context of Metabric data, which is of high quality only 20 patients out of 1992 are censored due to loss-in-follow-up. All other censored cases of type (3) are censored due to their entry the observation program later than 5 years before the end of the observation period (end-of-study). Note that our choice of removing patients of type (3) does not introduce any bias, as the time-of-entry of a patient in a study is considered independent of any other feature of the patient. As Metabric data are sufficiently numerous we can afford to neglect the patient of type (3) and still attain statistically significant results in most cases. In situations where data is not sufficient, one might want to adopt the full approach in Zupan et al. 2000 and assign a marginal probability of event occurrence estimated by the Kaplan–Meier method to patients of type (3).

### Statistics on patients features

Here we report the distribution of 17 patient features (continuous and categorical) for training, validation, and testing sets (after possible equalization in a therapy class). Categorical features in Table 10 are: intrinsic subtypes, type of breast surgery, histological subtypes, inferred menopausal status, HER2 SNP6 copy number gain/loss, laterality, intrinsic clustering, cohort of origin, ER status by immunohistochemical analysis, hormone receptors status, cellularity, lymph node status, tumor stage and tumor grade. Continuous features in Table 11 are age at diagnosis (in years), NPI (Nottingham prognostic index), and overall survival (in months). The repeated use of uniform random sampling ensures a high similarity in the distribution of the feature values across the train, validate and test sets.

**Table 10:** Distribution of patient categorical features over training, validation, and testing sets.

	Train		Validation		Testing	
**Lymph node**						
Num	326	–	186	–	200	–
NEG	113	34%	56	30%	67	33%
POS	213	65%	130	69%	133	66%
No data	4	–	4	–	2	–
**Stage**						
Num	240	–	141	-	155	–
1	50	20%	25	17%	27	17%
0	1	0%	0	0%	0	0%
3	27	11%	25	17%	25	16%
2	161	67%	91	64%	100	64%
4	1	0%	0	0%	3	1%
No data	90	–	49	–	47	–
**Grade**						
Num	323	–	185	–	197	–
1	19	5%	5	2%	9	4%
3	207	64%	120	64%	127	64%
2	97	30%	60	32%	61	30%
No data	7	–	5	–	5	–
**Subtype**						
Num	328	–	187	–	202	–
Normal	27	8%	15	8%	10	4%
Basal	75	22%	27	14%	32	15%
Her2	52	15%	27	14%	39	19%
LumB	67	20%	50	26%	42	20%
Claudin-low	37	11%	23	12%	29	14%
LumA	70	21%	45	24%	50	24%
No data	2	–	3	–	0	–
**Surgery**						
Num	326	–	186	–	200	–
MASTECTOMY	209	64%	118	63%	130	65%
BREAST-CONSERVING	117	35%	68	36%	70	35%
No data	4	–	4	–	2	–
**Histology**						
Num	330	–	190	–	202	–
IDC+ILC	11	3%	14	7%	10	4%
IDC-MUC	6	1%	6	3%	5	2%
ILC	25	7%	9	4%	15	7%
OTHER-INVASIVE	1	0%	1	0%	0	0%
OTHER	1	0%	0	0%	0	0%
IDC-MED	6	1%	2	1%	3	1%
INVASIVE-TUMOUR	3	0%	0	0%	1	0%
IDC-TUB	6	1%	3	1%	3	1%
DCIS	1	0%	0	0%	0	0%
IDC	270	81%	155	81%	165	81%
No data	0	–	0	–	0	–
**Menopause**						
Num	330	–	190	–	202	–
Pre	111	33%	56	29%	60	29%
Post	219	66%	134	70%	142	70%
No data	0	–	0	–	0	–
**Her2 SNP6**						
Num	330	–	190	–	202	–
NEUT	224	67%	131	68%	137	67%
LOSS	20	6%	8	4%	8	3%
GAIN	86	26%	51	26%	57	28%
No data	0	–	0	–	0	–
**Laterality**						
Num	306	–	181	–	189	–
r	140	45%	92	50%	87	46%
l	166	54%	89	49%	102	53%
No data	24	–	9	–	13	–
**Cluster**						
Num	330	–	190	–	202	–
4.5	34	10%	19	10%	21	10%
10	78	23%	36	18%	33	16%
1	24	7%	17	8%	10	4%
3	33	10%	22	11%	23	11%
2	8	2%	12	6%	8	3%
5	49	14%	27	14%	30	14%
4	17	5%	5	2%	12	5%
7	20	6%	11	5%	8	3%
6	10	3%	7	3%	10	4%
9	25	7%	16	8%	19	9%
8	32	9%	18	9%	28	13%
No data	0	–	0	–	0	–
**Cohort**						
Num	330	–	190	–	202	–
1	95	28%	58	30%	57	28%
3	115	34%	61	32%	73	36%
2	44	13%	29	15%	36	17%
5	27	8%	19	10%	14	6%
4	49	14%	23	12%	22	10%
No data	0	–	0	–	0	–
**ER IHC**						
Num	328	–	189	–	199	–
Neg	150	45%	65	34%	72	36%
Pos	178	54%	124	65%	127	63%
No data	2	–	1	–	3	–
**ER/HER status**						
Num	292	–	167	–	185	–
HER2+	53	18%	24	14%	31	16%
ER−/HER2−	86	29%	37	22%	43	23%
ER+/HER2–High-Prolif	82	28%	65	38%	65	35%
ER+/HER2–Low-Prolif	71	24%	41	24%	46	24%
No data	38	–	23	–	17	–
**Cellularity**						
Num	320	–	186	–	200	–
High	180	56%	92	49%	106	53%
Moderate	108	33%	70	37%	75	37%
Low	32	10%	24	12%	19	9%
No data	10	–	4	–	2	–

**Table 11:** Distribution of patient continuous features over training, validation, and testing sets.

	Train	Validation	Testing
**NPI**			
Num	330	190	202
Mean	4.49	4.56	4.54
Std dev	1.12	1.00	1.17
Median	4.14	5.02	5.03
Min	1.03	2.01	1.05
Max	6.36	6.26	6.12
**OS months**			
Num	330	190	202
Mean	103.24	110.35	112.99
Std dev	76.09	76.35	77.80
Median	88.88	102.00	98.40
Min	4.17	0.10	5.83
Max	337.03	301.23	322.83
**Age at diagnosis**			
Num	330	190	202
Mean	56.48	58.25	57.09
Std dev	13.29	14.36	12.69
Median	55.30	58.97	57.38
Min	28.29	26.72	21.93
Max	90.00	96.29	84.73

### Ethical statement

 Patients were not directly involved in the study.

## Supplementary Information

Supplementary Information 1.

## Data Availability

Data supporting the findings of this study are available form the *Github* repository https://github.com/MarcoPellegriniCNR/Coherent-Voting-Network-for-BC-prognosis.

## Code availability

Custom software and code availability is to be agreed via licensing contracts with National Research Council of Italy.
